# Capsule Endoscopy-Guided Diagnosis of Small Bowel Lymphoma Presenting as Protein-Losing Enteropathy: Complementary Role of Peripheral Blood Flow Cytometry

**DOI:** 10.3390/diagnostics16071006

**Published:** 2026-03-27

**Authors:** Mohammed Abdulrasak, Olof Axler, Balázs Kapás, Ervin Toth

**Affiliations:** 1 Department of Clinical Sciences, Lund University, 202 13 Malmö, Sweden; ervin.toth@med.lu.se; 2 Department of Gastroenterology, Skåne University Hospital, 205 02 Malmö, Sweden; 3 Department of Clinical Genetics and Pathology, Skåne University Hospital, 221 84 Lund, Sweden; olof.axler@skane.se; 4 Department of Medical Oncology, Skåne University Hospital, 221 85 Lund, Sweden; balazs.kapas@skane.se

**Keywords:** protein-losing enteropathy, capsule endoscopy, small bowel lymphoma, flow cytometry, marginal zone lymphoma, multimodal diagnostics, gastrointestinal lymphoma

## Abstract

Protein-losing enteropathy (PLE) is an uncommon and often underrecognized manifestation of lymphoproliferative disorders and may be difficult to diagnose when conventional gastrointestinal investigations are unrevealing. We present an 82-year-old woman with recurrent hospital admissions initially spanning six months for diarrhea, weight loss, peripheral edema, and persistent hypoalbuminemia. Initial upper gastrointestinal endoscopy was normal, and colonoscopy was deferred due to intercurrent infection. Despite extensive laboratory and radiologic evaluation, including routine biochemical testing and imaging, the etiology of PLE remained unclear. Peripheral blood flow cytometry subsequently identified a small kappa-restricted monoclonal B-cell population compatible with marginal zone lymphoma, later confirmed on bone marrow biopsy, raising suspicion for gastrointestinal involvement. Video capsule enteroscopy demonstrated diffuse erosive and ulcerative disease throughout the small intestine, providing an anatomical explanation for the patient’s protein loss. Following lymphoma-directed therapy, repeat capsule enteroscopy showed complete normalization of the small bowel mucosa. This case highlights the diagnostic value of combining peripheral blood flow cytometry and capsule endoscopy in unexplained protein-losing enteropathy, a rare and diagnostically challenging presentation of indolent lymphoma, and illustrates the role of capsule imaging in both disease localization and treatment monitoring. As a single-case report, these findings are not generalizable, and further studies are required to evaluate the broader applicability of this diagnostic approach.

**Figure 1:**
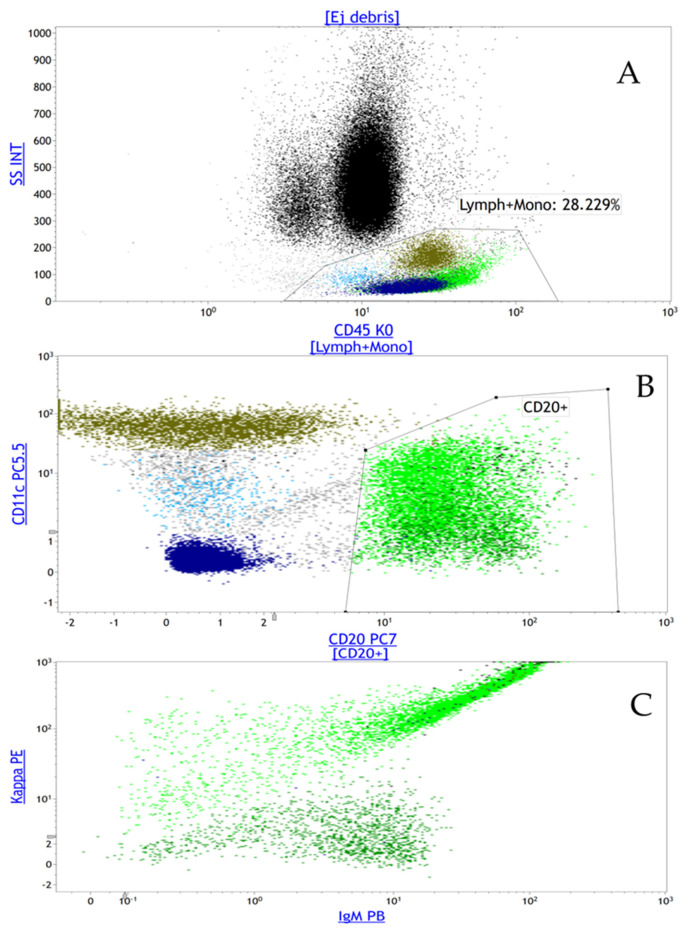
Peripheral blood flow cytometry demonstrating a kappa-restricted monoclonal mature B-cell population consistent with low-grade B-cell lymphoma. Multiparametric flow cytometric analysis of peripheral blood revealed that lymphocytes constituted 24% of total leukocytes, with B lymphocytes accounting for approximately 10% of circulating cells. Within this population, a distinct monoclonal B-cell subset (approximately 8% of total analyzed events) was identified. (**A**) Side scatter intensity (SS INT) gating demonstrates the lymphocyte and monocyte population comprising a total of 28.2% of the cells in the sample. (**B**) Immunophenotypic characterization shows expression of CD20 and CD11c within the abnormal B-cell population. CD20 confirms mature B-cell lineage, while CD11c positivity supports a marginal zone-type phenotype. The abnormal cells were negative for CD5, CD10, CD23, CD38, and CD103, thereby excluding chronic lymphocytic leukemia (typically CD5+/CD23+), follicular lymphoma (CD10+), mantle cell lymphoma (CD5+), and hairy cell leukemia (CD103+) [1]. (**C**) Light-chain analysis demonstrates strong kappa (κ) restriction (Kappa PE) with absence of lambda expression, confirming monoclonality. The clonal population also expresses surface IgM, consistent with a mature B-cell neoplasm of indolent phenotype. The overall immunophenotypic profile—CD19+, CD20+, CD11c+, surface IgM+, CD5−, CD10−, CD23−, CD103− with kappa light-chain restriction—is most compatible with a low-grade mature B-cell lymphoproliferative disorder, particularly marginal zone lymphoma, although lymphoplasmacytic lymphoma remains within the differential diagnosis. In the clinical context of persistent hypoalbuminemia and suspected protein-losing enteropathy, detection of this circulating monoclonal B-cell population constituted the first objective systemic evidence of lymphoma after inconclusive gastrointestinal investigations and prompted further targeted evaluation for extranodal involvement [2]. Prior investigations undertaken during the patient’s “diagnostic journey” spanning approximately 6 months and including routine laboratory testing, upper gastrointestinal endoscopy, and radiologic imaging were inconclusive and failed to identify an inflammatory, infectious, or structural cause of protein loss and hypoalbuminemia. The flow cytometry results illustrated above, however, provided a diagnostic clue that guided subsequent investigations, after six months of multiple investigations with negative or inconclusive results. The patient has a background history of hypertension and mild immune thrombocytopenia diagnosed approximately a decade prior to presentation.

**Figure 2:**
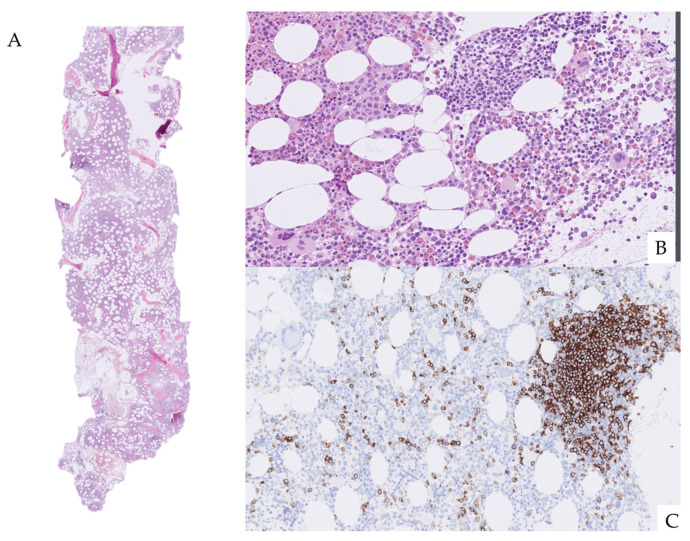
Bone marrow biopsy demonstrating nodular, interstitial, and intrasinusoidal infiltration by CD20-positive small B lymphocytes consistent with indolent B-cell lymphoma. (**A**) Low-power view of a 14 mm bone marrow core biopsy demonstrating preserved trabecular architecture and overall cellularity of approximately 40% within the upper expected range for age. Several small, discrete nodular lymphoid aggregates are visible within the marrow space. (**B**) Hematoxylin and eosin (H&E) staining at higher-power magnification reveals small mature lymphocytes forming nodular and partially interstitial infiltrates, without significant atypia or blast morphology. Background hematopoiesis is preserved, with maintained myeloid and erythroid maturation. No abnormal plasmacytosis or blast proliferation is identified. (**C**) Immunohistochemical staining at higher-power magnification for CD20 demonstrates strong positivity of the lymphoid infiltrates, confirming B-cell lineage. The infiltrative pattern includes nodular aggregates as well as interstitial and intrasinusoidal distribution of CD20-positive cells. This pattern is characteristic of marrow involvement by low-grade mature B-cell lymphoproliferative disorders, including marginal zone lymphoma. No aberrant expansion of plasma cells or high-grade transformation features are observed. In the clinical context of peripheral blood kappa-restricted monoclonal B-cell population and persistent protein-losing enteropathy, these findings confirm systemic indolent B-cell lymphoma with bone marrow involvement [3]. While marrow infiltration establishes disseminated disease, it does not localize the source of gastrointestinal protein loss. Therefore, direct small bowel evaluation was required to determine the presence and extent of extranodal intestinal involvement.

**Figure 3:**
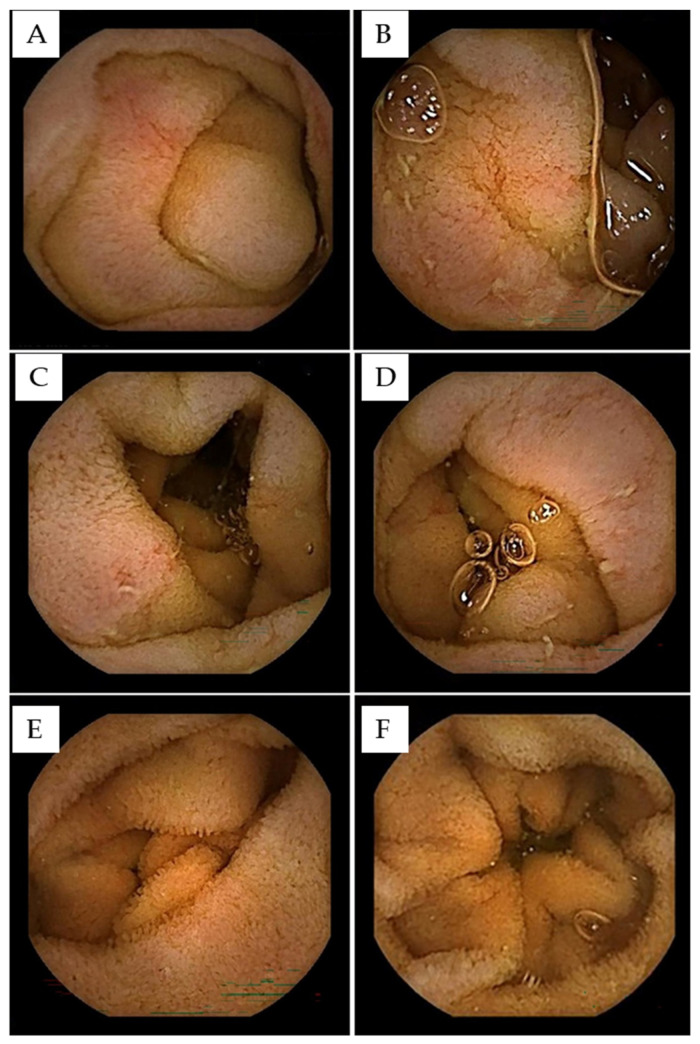
Video capsule enteroscopy demonstrating diffuse proximal and mid-small bowel mucosal abnormalities suggestive of lymphomatous involvement of the small bowel. Representative still images (**A**–**D**) from baseline video capsule enteroscopy (VCE) demonstrating diffuse small bowel mucosal abnormalities predominantly involving the proximal and mid-small intestine. The mucosa appears mildly but diffusely edematous with blunted villous architecture. Multiple erosions and small ulcerations are observed, affecting broad segments of the proximal jejunum (**A**,**B**) and extending into the mid-small bowel (**C**,**D**). No strictures or active bleeding are identified. Similar capsule endoscopy findings in small bowel lymphoma have been described, including diffuse mucosal edema, erosions, and ulcerations without discrete mass formation, reflecting mucosa-predominant infiltrative disease [4,5]. Differential diagnostic considerations included inflammatory bowel disease (particularly Crohn’s disease), drug-induced enteropathy, infectious enteritis, lymphomatous involvement of the small bowel and other non-mucosal causes of protein-losing enteropathy such as intestinal lymphangiectasia [6,7,8]. However, the absence of deep linear ulcerations, cobblestoning, skip lesions, or stricturing features argues against classic Crohn’s disease [9,10]. In the context of a confirmed systemic kappa-restricted low-grade B-cell lymphoproliferative disorder, the diffuse erosive pattern with villous blunting was considered highly suggestive of small bowel lymphomatous infiltration contributing to the patient’s protein-losing enteropathy. Capsule endoscopy, while highly sensitive for mucosal abnormalities in multiple pathologies, does not allow for histological sampling. Therefore, interpretation of VCE findings requires integration with clinical, hematologic and follow-up VCE imaging. Follow-up after lymphoma-directed therapy demonstrated clinical improvement, supporting treatment response. Follow-up VCE performed eight months later showed complete normalization of the entire small bowel mucosa with resolution of erosions and reconstitution of normal villous structures (panels (**E**,**F**)). This case highlights the complementary diagnostic value of peripheral blood flow cytometry and VCE in patients with unexplained protein-losing enteropathy. Flow cytometry may provide an early clue towards an underlying lymphoproliferative disorder. VCE—having undergone significant technological advancement—has a key role in evaluating small bowel pathologies, including neoplastic and inflammatory conditions [11,12], and aids in defining disease extent and longitudinal monitoring of gastrointestinal involvement.

## Data Availability

The original contributions presented in the study are included in the article, further inquiries can be directed to the corresponding author.
